# Supplementary material for: A free energy landscape analysis of resistance fluctuations in a memristive device
Source: Nat Mater. 2026 Jan 30;25(4):643–50. doi: 10.1038/s41563-026-02487-9 (PMC13046472; doi:10.1038/s41563-026-02487-9)
Supplement: Supplementary file 1 — Supplementary Figs. 1–3 and Discussion. [file 41563_2026_2487_MOESM1_ESM.pdf]

nature materials

Supplementary information https://doi.org/10.1038/s41563-026-02487-9

A free energy landscape analysis of resistance fluctuations in a memristive device

In the format provided by the authors and unedited

Contents

1 Model checking with pseudo-residuals 1

2 Correlations in attempt frequency and activation energy 2

3 Correlations in the amorphous volume 3

1 Model checking with pseudo-residuals

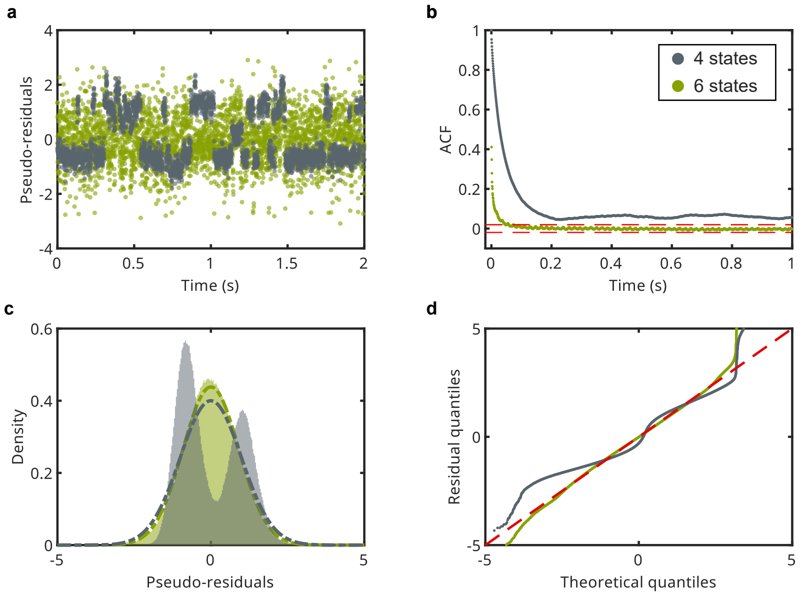

Supplementary Fig. 1 Pseudo-residuals of a four- and six-state model: a Excerpt of the time-series of pseudo-residuals **b** Dotted lines are sample autocorrelation function of the pseudo-residuals. The red dashed lines indicates the approximate 95% confidence bounds for a white-noise (zero-autocorrelation) sequence, computed as $\pm 1.96/\sqrt{n}$, where $n = 10000$ is the length of the pseudo-residual time series. **c** Histogram of pseudo-residuals together with fit of normal distribution **d** Normal quantile-quantile plot of pseudo-residuals

To check the validity of a hidden Markov model we calculate the (ordinary) pseudo-residuals, as described in Zucchini et al. [1]. While the calculation of pseudo-residuals is slightly more involved compared to the residuals in more standard regression analysis, they serve the same purpose in assessing the goodness of a fit [2, 3]. That is, a model can describe the data if the pseudo-residuals are approximately normally distributes and the time series is featureless, with quickly decaying autocorrelation. Supplementary Fig. 1 shows the pseudo-residuals of a hidden Markov model fit with four and six states to the same data set.

The pseudo-residuals of the six-state model are approximately normally distributed and exhibit little autocorrelation, suggesting that the model can indeed describe the

measured noise dynamics. The occasional outlier could be "mopped up" with additional states, without significantly altering the transition rate between any of the six main states. In contrast, four states are clearly insufficient to describe the data. We found that Akaike or Bayesian information criteria tend to drastically overestimate the number states for our data, compared to an estimation based on pseudo-residuals.

2 Correlations in attempt frequency and activation energy

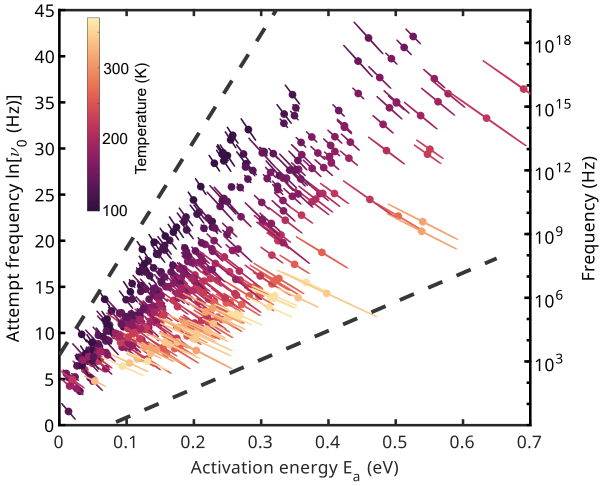

Supplementary Fig. 2 Correlations effects: Scatter plot of attempt frequency and activation energy extracted from fits of the form $W_{ij}(T) = \nu_0 \exp(-E_a/k_\mathrm{B}T)$ (linear regression of $\ln W_{ij}$ vs. $1/T$). Each marker shows the best-fit pair $(E_a, \ln \nu_0)$ for a given directed state-to-state transition rate identified in the hidden-Markov analysis; the colour encodes the mean temperature $\langle T \rangle$ of the temperature interval over which that transition was observable. Ellipses denote the 95% joint parameter confidence region for $(E_a, \ln \nu_0)$ obtained from the regression covariance matrix. The number of temperature points entering each Arrhenius fit is n (amorphous state- and temperature-dependent, $n > 5$). The plotted uncertainty therefore reflects fit-parameter uncertainty within the regression model. The narrow ellipses arise from the strong covariance between E_a and $\ln \nu_0$. Grey dashed lines indicate the experimentally accessible observation window.

The finite time resolution and measurement duration of the experiments induce correlations in the Arrhenius pre-exponential factor and the activation energy. That is, for an energy barrier to be observed, the corresponding transition rate must cross the frequency versus inverse temperature observation window, as shown for example in Fig. 2c of the main text. Supplementary Fig. 2 plots the natural logarithm of the resulting attempt frequencies versus the corresponding activation energies. All observed barriers can only lie in the region between the two dashed lines, defined by

$$\ln\left[\nu_0\left(E_a\right)\right] = \ln \nu_{\mathrm{max,min}} + \frac{E_a}{k_B\, T_{\mathrm{min,max}}}. \tag{1}$$

The upper dashed line is given by the $\nu_{\text{max}} \approx 1\,\text{kHz}$ bandwidth of the experiment and the $T_{\text{min}} = 100\,\text{K}$ low end of the temperature range, and the lower dashed lines by $\nu_{\text{min}} \approx 0.1\,\text{Hz}$ and $T_{\text{max}} = 370\,\text{K}$. The temperature trends in Supplementary Fig. 2 demonstrate that the limits for observing a specific barrier over a temperature interval are more restrictive. Only by measuring across a wide temperature range can we resolve certain combinations of attempt frequency and activation energy and illuminate different landscape barriers.

3 Correlations in the amorphous volume

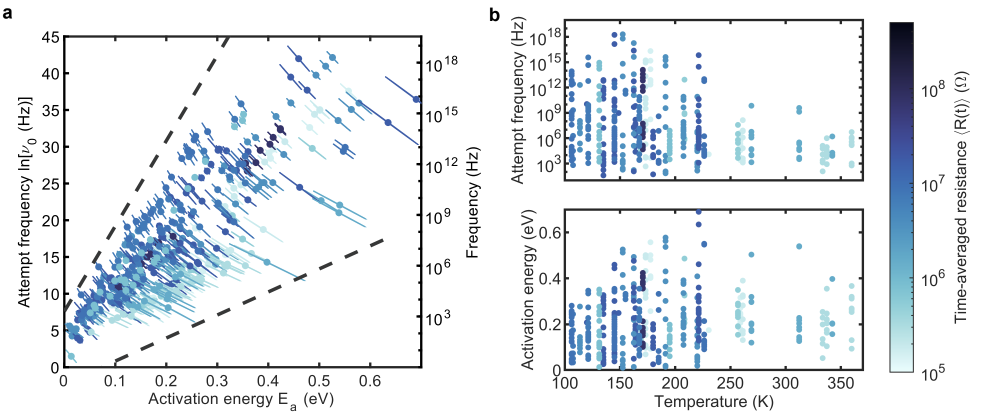

Supplementary Fig. 3 Role of the amorphous volume: a Scatter plot of attempt frequency and activation energy extracted from fits of the form $W_{ij}(T) = \nu_0 \exp(-E_a/k_\text{B}T)$ (linear regression of $\ln W_{ij}$ vs. $1/T$). Each marker shows the best-fit pair $(E_a, \ln\nu_0)$ for a given directed state-to-state transition rate identified in the hidden-Markov analysis. Instead of temperature, the time-averaged resistance of the GeTe cell is colour coded. As in Supplementary Fig. 2 above, ellipses denote the 95% joint parameter confidence region for $(E_a, \ln\nu_0)$ obtained from the regression covariance matrix. The number of temperature points entering each Arrhenius fit is n (amorphous state- and temperature-dependent, $n > 5$). The plotted uncertainty therefore reflects fit-parameter uncertainty within the regression model. The narrow ellipses arise from the strong covariance between E_a and $\ln\nu_0$. Grey dashed lines indicate the experimentally accessible observation window. **b** Pairs of attempt frequencies and activation energies plotted against the average of the temperature interval where the corresponding states were observed. Instead of signifying the different measurement series as in Figure 1b of the main text, the time-averaged resistance of the GeTe cell is color coded.

While the combinations of attempt frequencies and activation energies, that can be observed experimentally, are governed by the temperature (Supplementary Fig. 2), we do not find such clear correlations with the time-averaged resistance, i.e. the dimensions of the probed glass in the GeTe cell (Supplementary Fig. 3). Within a narrow temperature window, i.e. comparable resistivities, cells with resistances ranging from $100\,\text{k}\Omega$ to $100\,\text{M}\Omega$ exhibit a comparable spread in attempt frequencies and activation energies. At temperatures above 250 K the system can sample a larger set of activation barriers. The glass volume must therefore be reduced more drastically in order to observe discrete resistance levels in the time trace.

References

[1] Zucchini, W., MacDonald, I.L., Langrock, R.: Hidden markov models for time series: An Introduction Using R, Second Edition. Chapman & Hall (2016)

[2] Langrock, R., Hopcraft, J.G.C., Blackwell, P.G., Goodall, V., King, R., Niu, M., Patterson, T.A., Pedersen, M.W., Skarin, A., Schick, R.S.: Modelling group dynamic animal movement. Methods in Ecology and Evolution **5**(2), 190–199 (2014) https://doi.org/10.1111/2041-210X.12155

[3] McClintock, B.T., Langrock, R., Gimenez, O., Cam, E., Borchers, D.L., Glennie, R., Patterson, T.A.: Uncovering ecological state dynamics with hidden markov models. Ecology Letters **23**(12), 1878–1903 (2020) https://doi.org/10.1111/ele.13610
